# 75 years’ journey of malaria publications in English: what and where?

**DOI:** 10.1186/s12936-024-04992-1

**Published:** 2024-06-02

**Authors:** Nimita Deora, Sonalika Kar, Veena Pande, Abhinav Sinha

**Affiliations:** 1 ICMR-National Institute of Malaria Research, New Delhi, India; 2 Kumaun University, Nainital, India; 3 Academy of Scientific and Innovative Research, New Delhi, India

**Keywords:** Malaria, Publications, Journals, Countries, Subject

## Abstract

Malaria has inflicted serious morbidity and mortality across the globe. The major brunt of the disease has been on African, South-East Asian and South American countries. Proportionally, malaria has attracted global research priorities and this is evident from the number of publications related to malaria from across the globe, irrespective of its endemicity. However, formal and exhaustive analyses of these ‘malaria publications’ are rarely reported. The systematic review and secondary data analyses were done to retrieve information on what has been published on malaria, where is it published, and which countries are major contributors to malaria research.

The study presents malaria publications from 1945 to 2020 retrieved using three databases: Web of Science^™^, Embase^®^ and Scopus^®^. Exported data were examined to determine the number of publications over time, their subject areas, contributions from various countries/organizations, and top publishing journals.

The total number of published records on malaria ranged from 90,282 to 112,698 (due to three different databases). Based on the number of publications, USA, UK, France, and India were identified as the top four countries. Malaria Journal, American Journal of Tropical Medicine & Hygiene, and PLoS One were the most preferred journals, whereas the University of London (Institutions other than LSHTM), the National Institute of Health, the London School of Hygiene and Tropical Medicine, and the University of Oxford appeared to be the top contributing organization.

A disproportional contribution to malaria research was observed with non-malaria endemic countries making the largest contribution. Databases differed in their output format and needed standardization to make the outputs comparable across databases.

## Background

One of the most effective ways to translate research to fellow researchers includes the publication of research in peer-reviewed journals. The research must be published and/or documented and disseminated, otherwise, it defies the scientific purpose and ethics [1]. Timely realization of the benefits of costly medical research is a global concern [2, 3] and publication-delays are viewed as a waste of limited resources and a potential loss of patient benefit, particularly for diseases such as malaria which is targeted for elimination by the year 2030. Therefore, the number of scientific journal publications on malaria per unit of time is a proxy indicator of its research importance and political commitment. Undoubtedly, it is important to analyse the trends of malaria publications over the years, including the journals that publish malaria, the subject areas within malaria that are predominant, the countries that publish the most malaria-related papers. The most common way to perform such publication analysis is through the use of online databases, some of which are free to use and some are available on a subscription basis.

Although there are several databases available, each database is unique in terms of both its content and ease of use. Some databases focus mainly on the content type (articles, images, videos, full-text pieces of literature, or more) whereas many databases focus on only one specific subject area or disciplinary field (valuable for advanced researchers). Some databases vary in their access to content (links directly to full-text and other databases provide basic article information) whereas some are equipped with different search features.

Although a few bibliometric studies on malaria are reported, they were either restricted to a particular geography (Latin America [4]; China [5, 6]; India [7, 8]; Malawi [9]; Portugal [10]; Central African Republic [11]), a particular period or a specific context related to malaria (artemisinin [12, 13]; malaria vaccines [14]; malaria in pregnancy [15]; malaria vector resistance [16]; anti-malarial drug resistance [17]; citations [18, 19]) or to a wider term such as mosquito-borne disease [20] or parasitology [21]. There was only one article that analysed global research on malaria [22] but it was focused on *Plasmodium vivax* and the data were retrieved only from the Scopus^®^. The current analyses were thus done to systematically showcase these publication trends in malaria by using 3 important databases that are said to retrieve more than 95% of available information: Web of Science^™^, Scopus^®^, and Embase^®^ [23].

## Methods

This was a systematic review and secondary data analysis of published literature on malaria. A one-time search was conducted across three major databases Web of Science^™^ (WOS), Embase^®^ (EMB), and Scopus^®^ (SCO) on May 09, 2021, using the boolean operator “Plasmodium or Malaria”. WOS was explored through the available ‘basic search’ option with a secondary selection on ‘topic’. EMB was searched using the ‘quick search’ option where no further option was available for restricting the search term to title or abstract whereas SCO allowed using the ‘search documents’ tab for “Plasmodium or Malaria” in article title, abstract, or keywords. No time-span filter was applied. Meeting abstracts, proceeding papers, notes, news items, corrections, reprints, biographical items, retracted publications, conference reviews, erratum, obituaries (mentioned as tombstones), and reports published other than in the English language were excluded from the final analyses.

The databases were explored during a free-trial access period provided to ICMR-National Institute of Malaria Research, New Delhi. The outcomes from 3 databases were available in two formats—entire comprehensive information (raw data) of all the published reports and pre-classified data (already analysed data file on limited parameters). SCO did not permit the export of raw data in the trial period; hence only pre-classified data was exported from SCO whereas both raw- and pre-classified data were obtained from WOS and EMB.

Exported data were analysed to address the number of publications over time, their subject areas, contributions from different countries/organizations, and names of publishing journals. Because subject areas were not uniform across the databases, the subject areas were re-grouped into broader categories to make the data presentable and comparable across the databases. The top-ten ranked countries, organizations, journals, and subject areas were identified using pre-classified data.

The contribution from different countries was calculated based on the affiliations of authors (all). However, if a single study has multiple affiliations from the same country, the results were counted as one as the databases applied a deduplication filter which removes duplicates. In contrast, if a single article is affiliated with different countries, each country’s contribution is counted separately. The same applies to institutional contributions. To examine country-specific contribution, the numerator and denominator were the number of published records with affiliation from that specific country and the total number of published records with affiliation from any country, respectively (as obtained from pre-analysed data).

Here it is important to note that, the denominator (obtained from pre-analysed data) varies according to the parameter analysed. For instance, the denominator in country-wise analysis was greater than the denominator of year-wise analysis since each record was counted once in year-wise analysis, while records might have been counted more than twice in country-wise analysis if they have affiliations from more than one country.

Apart from identifying top-ranked countries, the contribution of countries was reanalysed based on their respective population. The total population of the countries in 2021 (https://datacommons.org/place) was used as a denominator for calculating publications per million population using the two databases SCO and WOS. The top 10 countries with the highest number of publications on malaria and the top 10 countries with the highest publications per million populations were listed from the above data. To identify all unique journals that published malaria over time, journal details from all EMB, SCO, and WOS databases were merged, and the total number of journals was determined after removing duplications.

For determining the number of malaria publications every year from the top ten journals, the raw data from WOS was only used because it offered the longest historical data (since 1945). The raw data obtained from WOS was cleaned and sorted year-wise for each of the top ten journals to calculate the year-wise number of publications of that particular journal.

To determine the top ten subjects under which malaria-related literature was published over time, the number of published records in a specific subject and the number of records published in any subject were used as the numerator and denominator, respectively. Similarly, to calculate the organization-specific contribution to malaria publications over time, the number of published records with affiliation to a specific organization and the number of published records with affiliation to any organization were used as the numerator and denominator, respectively. These numerators and denominators were obtained from pre-analysed data of selected databases.

Furthermore, to ascertain the trend of malaria publications with malaria burden, the estimated number of malaria cases [24] was recorded and plotted against the year-wise malaria publications from the top ten journals. Only WOS was used for this analysis because this was the only database that contained data from 1945.

## Results

**Fig. 1:**
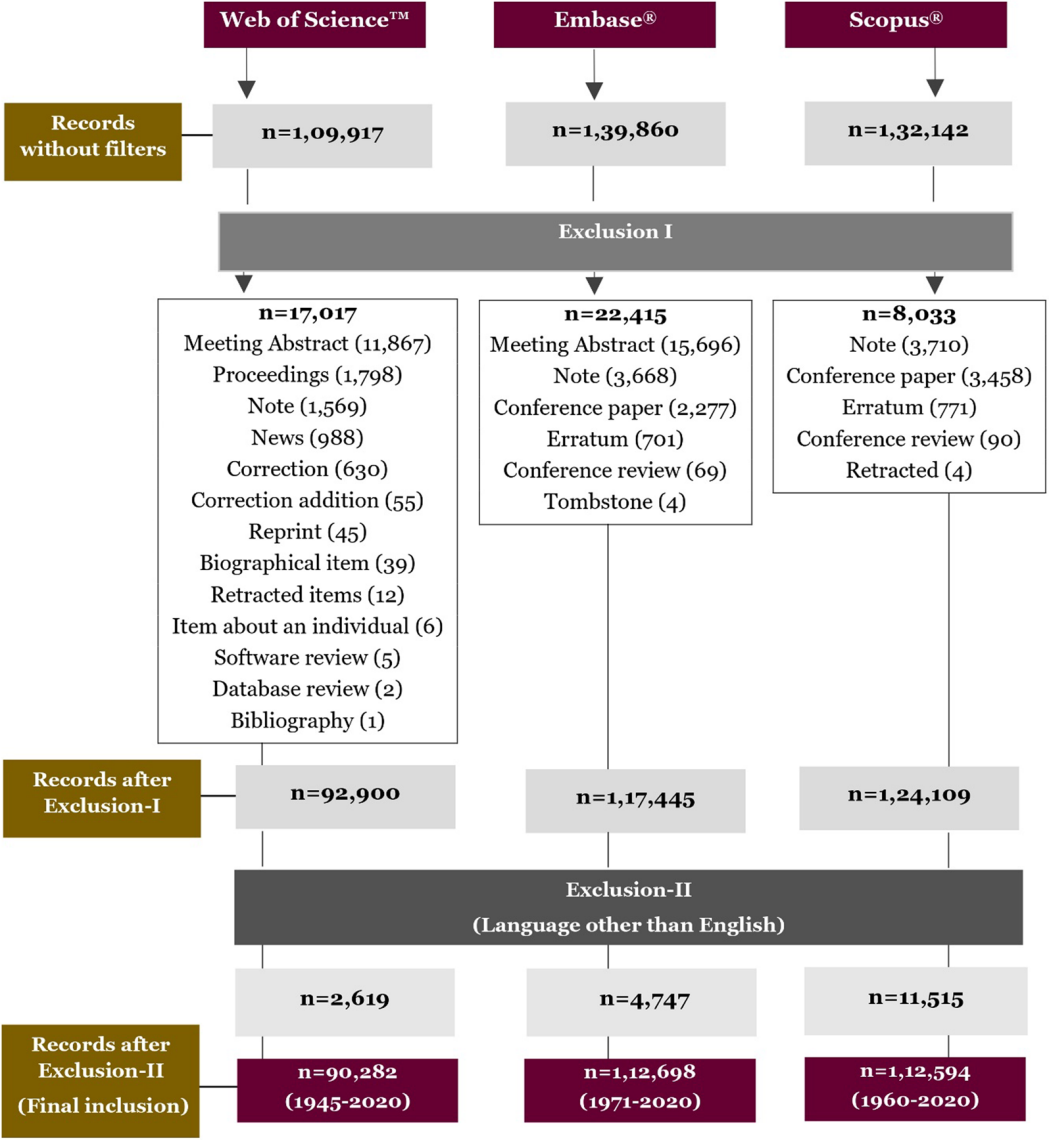
Search strategy for data extraction from Web of Science^™^, Scopus^®^ and Embase^®^. The figure depicts the search strategy for Web of Science^™^, Scopus^®^, and Embase^®^ along with all the exclusions used in each step, in order to generate results

The total number of published literature (and temporal coverage in years) on malaria retrieved from WOS, EMB and SCO were 90,282 (1945–2020; 75 years), 112,698 (1971–2020; 49 years) and 112,594 (1960–2020; 60 years) respectively (Fig. 1).

**Fig. 2:**
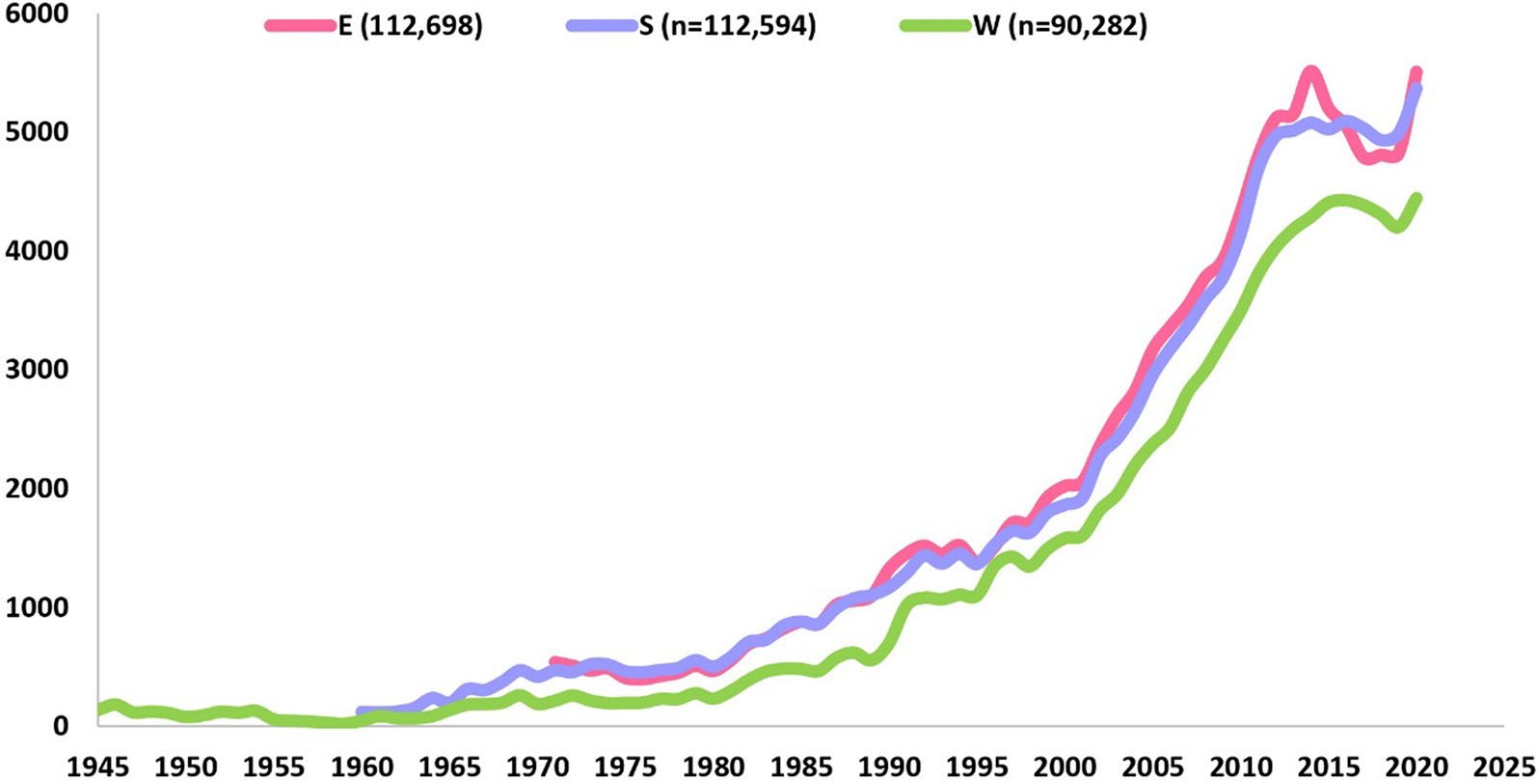
Number of annual malaria publications obtained from three different databases. The graph displays the evolution of the number of annual publications on malaria over time obtained from three databases Embase^®^ (E), Scopus^®^ (S) and Web of Science^™^ (W). Here, x-axis and y-axis display year and number of publications, respectively

Records indicate that the first documents were published in 1945 (obtained from WOS). The other two search engines, SCO and EMB, provided records of malaria-related documents that were initially published in 1960 and 1971, respectively (Fig. 2). The highest number of annual publications (n = 5517) was recorded in 2014, followed by n = 5509 in 2020 (EMB).

**Fig. 3:**
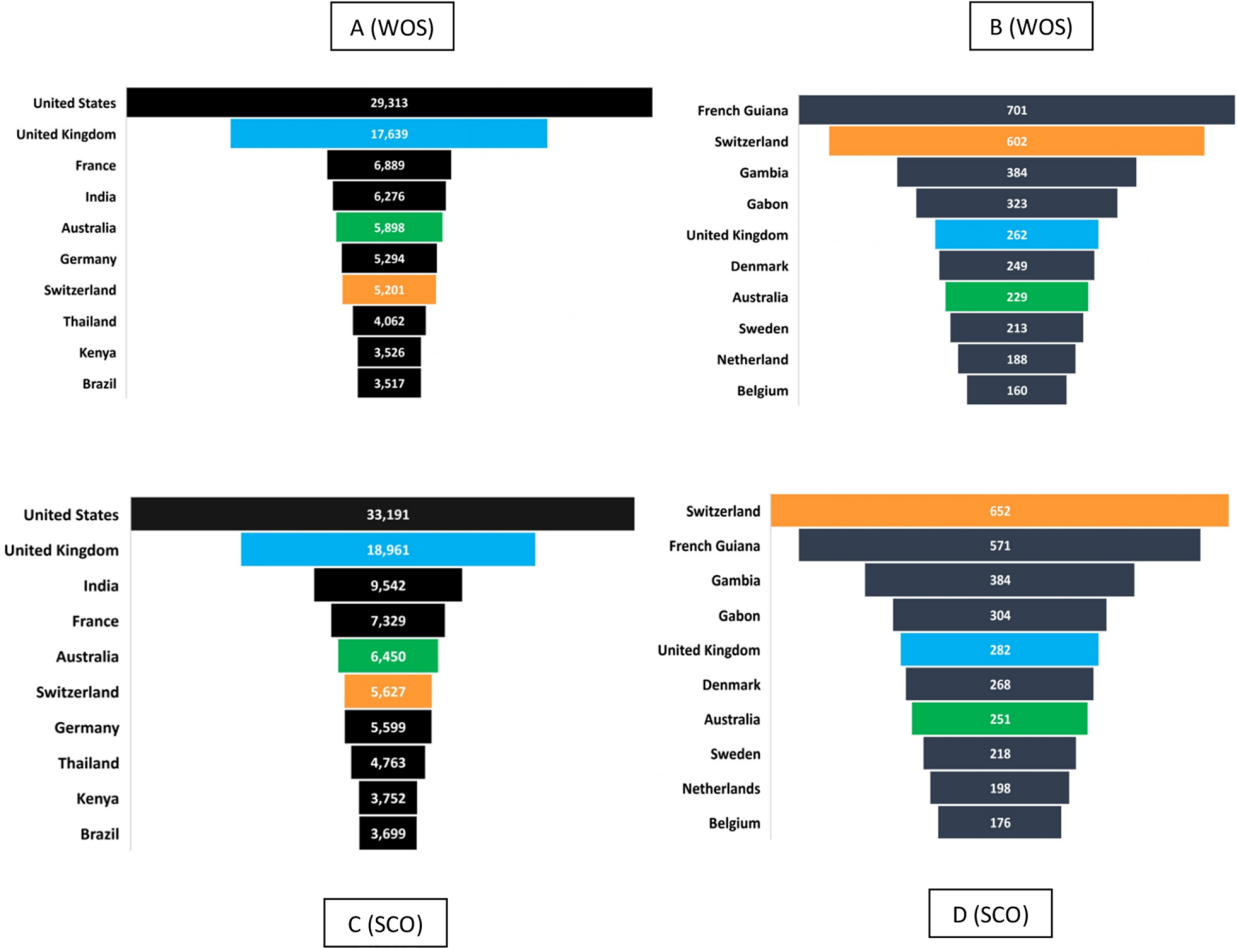
**A**–**D** Top ten countries based on published records with affiliation from that particular country (**A**, **C**) and publications per million populations (**B**, **D**). Figures A and C were created by plotting the number of publications with affiliations from a specific country from 1945 to 2020, as obtained from WOS and SCO, respectively. Figures B and D show the top ten countries based on their publications per million populations as determined by WOS and SCO, respectively. The total population of the countries in 2021 was used as a denominator in this calculation to calculate publications per million population of a specific country. Countries that consistently appeared in the top ranked list were colored the same in both figures (**A**–**D**) to emphasize their position. For UK in **D**, the number of publications from England, Scotland, and Wales was pooled. The information for Northern Ireland was not available in Scopus

Between 1945 and 2020, of all published records (with affiliation from any country) obtained from WOS (n = 150,239), the USA published 20%, followed by the UK (12%) and France (5%) (Fig. 3A). Despite India being the most affected countries by malaria (outside Africa), it contributed only 4%, which is five times less as compared to the USA. Similar trends were observed for SCO (n = 180,486) with the USA contributing the most (18%), followed by the UK (11%) whereas India contributed 5% during 1960–2020 (Fig. 3C). Other high and low-income countries published between 0.01 and 2% in both databases. However, when the publication data was standardized by the country-specific population, French Guiana, Switzerland, The Gambia, Gabon, and UK were the top 5 countries publishing on malaria as per WOS and SCO but their ranks differed between the databases as shown in Fig. 3B, D. It is to be noted that Switzerland, UK, and Australia were the only 3 countries that retained their slots in the top 10 countries publishing the most in malaria in both WOS and SCO. It should be noted that, despite being a malaria non-endemic country, the US is the largest contributor with 18–20% of the total published records (with affiliation from any country), with the remaining individual countries falling below 11%. The top ten countries contribute between 2 and 20% each. Based on data obtained from various databases, the least contributing countries were also identified which contribute to < 1%.

**Fig. 4:**
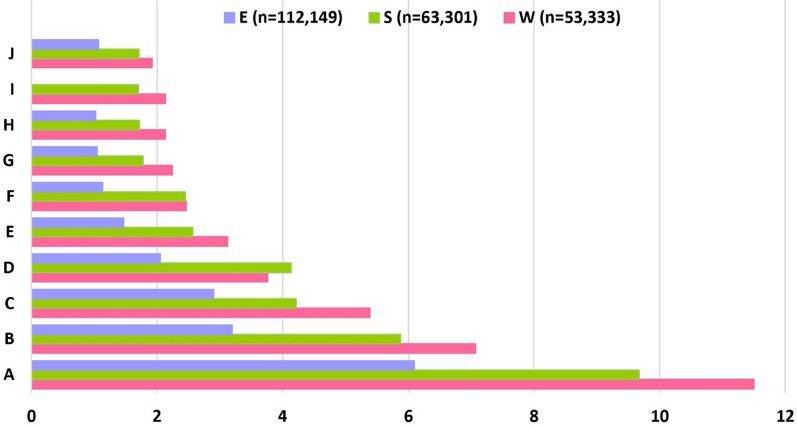
Journal-wise analysis of malaria related published records over the years. The figure represents the percentage contribution of the top 10 journals ranked from one (highest; A) to ten (lowest: J) in which various malaria related records were published during 1945–2020. Here, x-axis represents percentage of published records and y-axis represents journals coded by alphabets (A–J). Records from Embase^®^ (E), Scopus^®^ (S) and Web of Science^™^ (W) have been shown in blue, green, and pink respectively. **A**: Malaria Journal; **B**: American Journal of Tropical Medicine and Hygiene; **C**: PLoS One; **D**: Transactions of the Royal Society of Tropical Medicine and Hygiene; **E**: Molecular and Biochemical Parasitology; **F**: The Lancet; **G**: Infection and Immunity; **H**: Antimicrobial Agents and Chemotherapy; **I**: Experimental Parasitology; **J**: Journal of Infectious Diseases

**Fig. 5:**
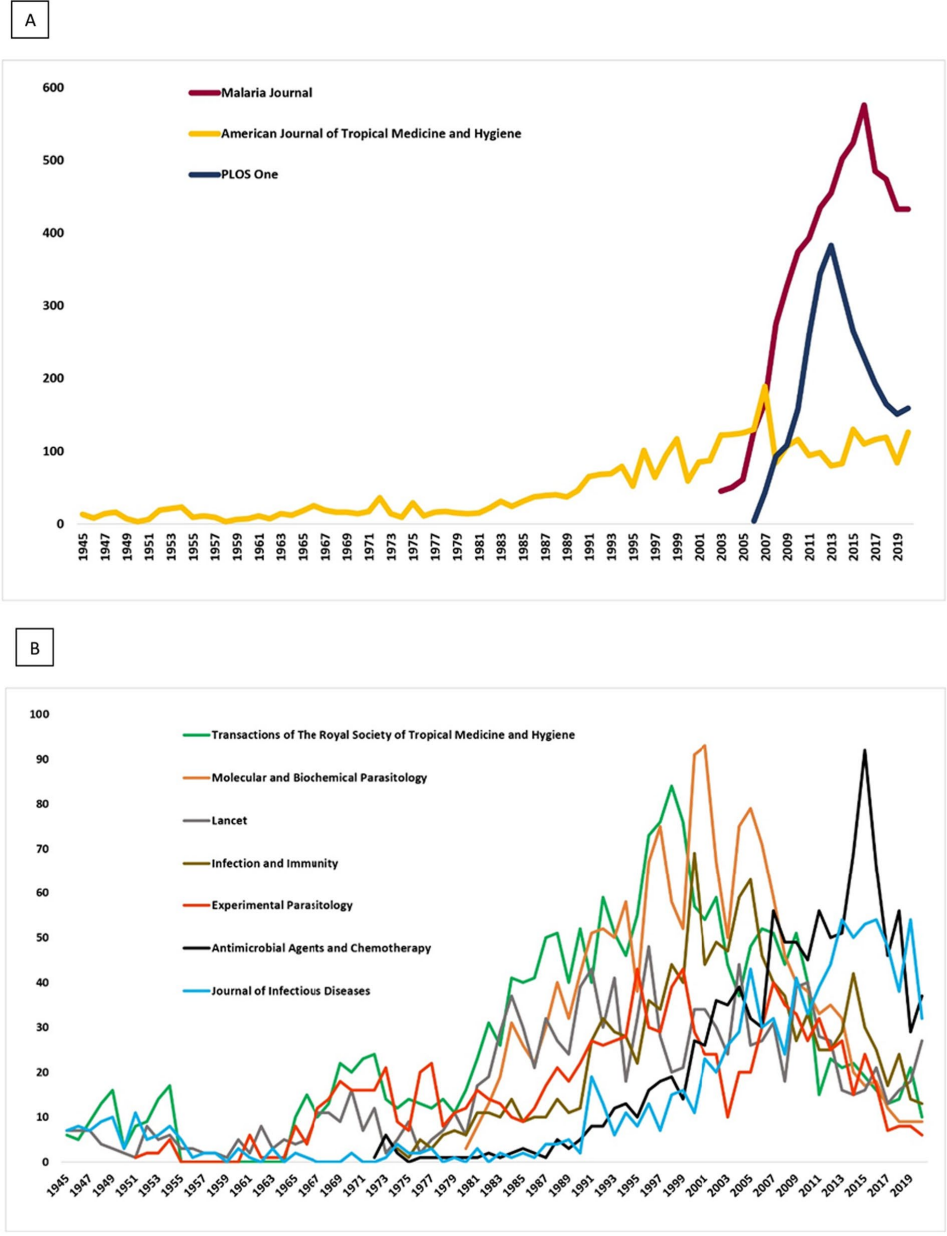
**A**, **B** Performance of the most active journals ranked from one to ten (on the basis of number of publications) between 1945 and 2020. **A** depicted the top three journals; Malaria Journal, AJTMH, and PLOS One, while **B** depicted the remaining seven journals. The Y axis represents the number of publications, while the X axis represents the year of publication. It is to be noted that the launch year of all journals was not the same

All the paper cited in the three databases came from 1811 different journals. The largest number of papers were published in the Malaria Journal as seen from the trend of publications (Fig. 4) in various journals indexed by WOS (n = 6133; 11.5%), SCO (n = 6123; 9.7%) and EMB (n = 6835; 6.1%). The top five preferred journals by researchers included Malaria Journal, American Journal of Tropical Medicine and Hygiene, PLoS One, Transactions of the Royal Society of Tropical Medicine and Hygiene, and Molecular and Biochemical Parasitology (Figs. 4, 5). The number of records published in the top ten journals in WOS, SCO and EMB was 22,256 (42%), 22,675 (36%), and 23,545 (21%), respectively (Fig. 5).

**Fig. 6:**
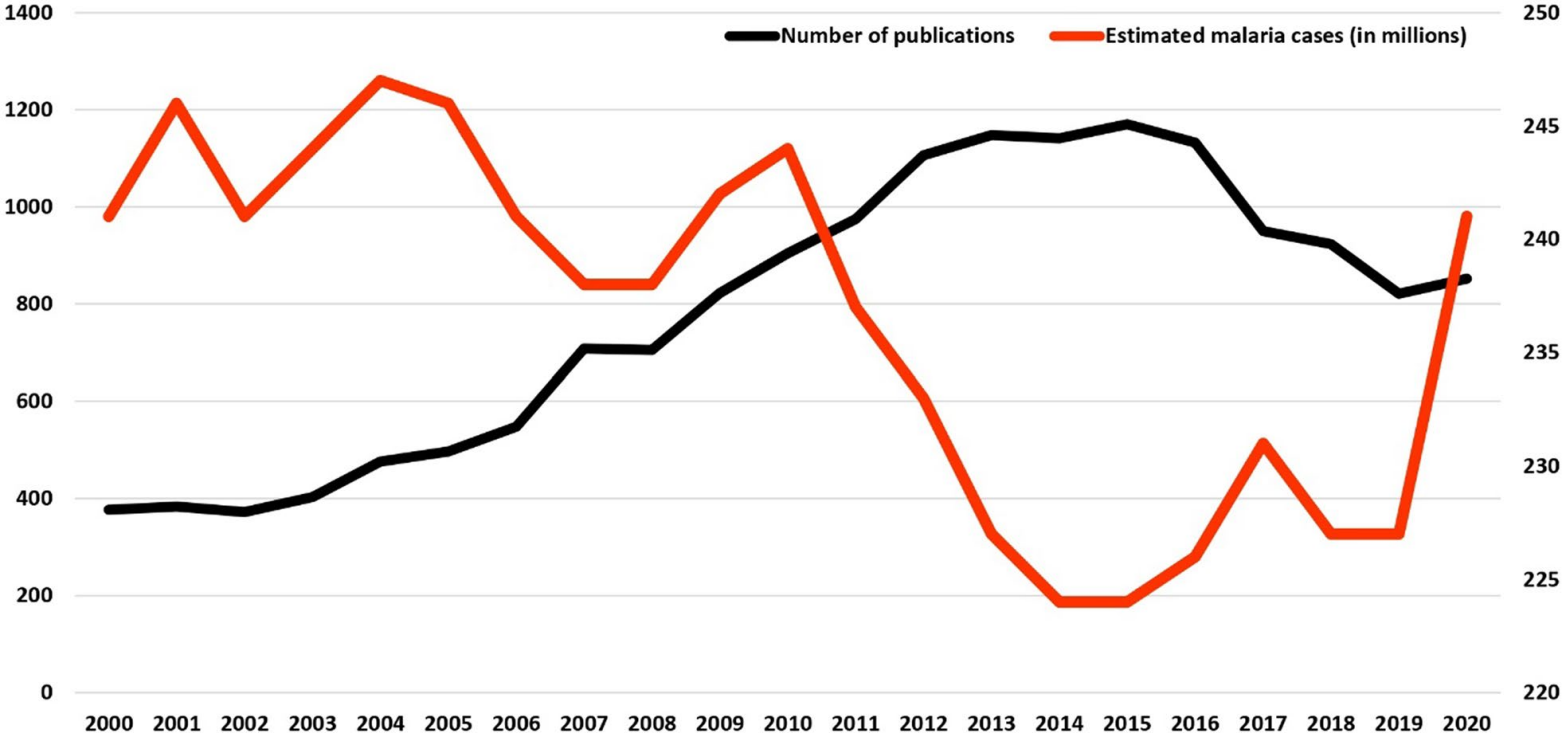
Correlation between the number of publications and the number of estimated malaria cases worldwide from 2000 to 2020. The primary Y-axis (0–1400) represents the total number of publications (black line) from the top 10 journals whereas the second Y-axis (220–250) shows the global estimated malaria cases in millions (red line)

Further, plotting published malaria records versus estimated global malaria cases revealed a steep fall in estimated malaria cases after 2010, which was mirrored in global malaria publications across all three databases between 2015 and 2020 (Fig. 6).

**Fig. 7:**
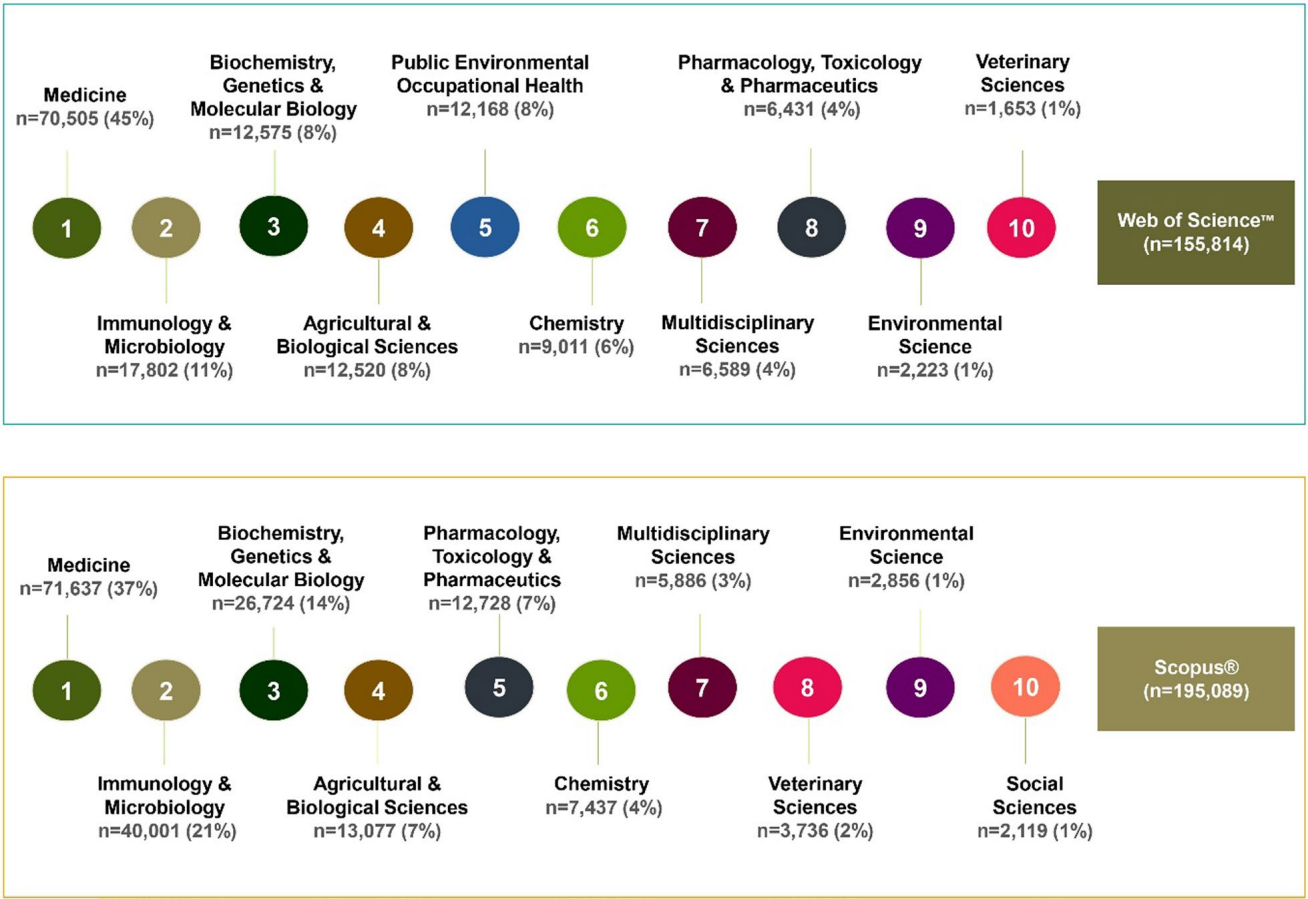
Subject-wise analysis of malaria related published records over the years. The figure depicts the top ten subjects (as determined by the Web of Science^™^ & Scopus^®^ databases) under which malaria-related literature were published over time. A specific subject has been highlighted with a specific colour in both databases. Subjects have been listed from one to ten in descending order based on the number of publications. Here, ‘n’ represents number of records published in a specific subject

It is clear from Fig. 7 that more than one-third of the total records were related to medicine (WOS 45%; SCO 37%). Here, it is to be noted that to get uniform data from two different databases, subjects were grouped into one category if they were initially found scattered across sub-categories. Other relevant subjects under which malaria-related records were published included immunology and microbiology, biochemistry, genetics and molecular biology, agricultural and biological sciences, pharmacology, toxicology, and pharmaceutics and public, environmental and occupational health. Further, such pre-classified data was not available in EMB and hence was not analysed for this particular category.

**Fig. 8:**
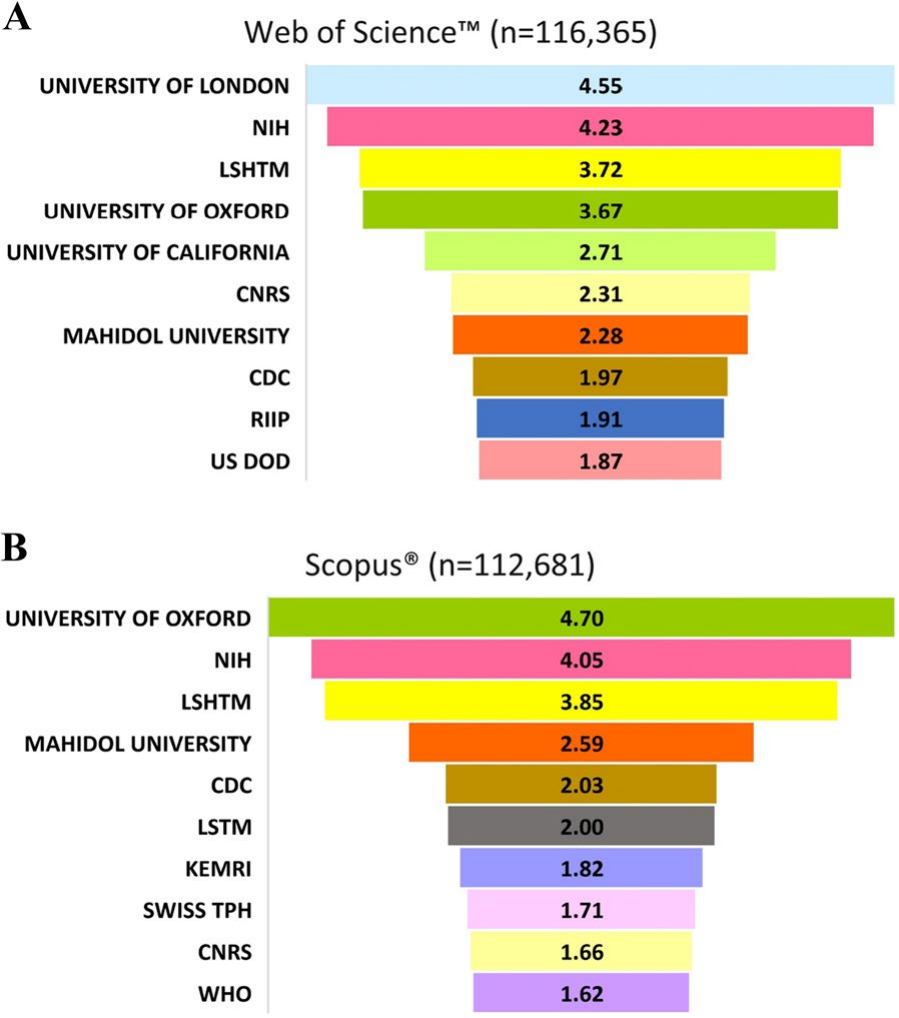
**A**, **B** Highest contributing organizations to the global malaria research. This figure depicts the most active scientific organizations dedicated towards malaria research based on the data obtained from Web of Science^™^ (**A**) & Scopus^®^ (**B**). Here, top ten contributing organizations have been arranged in descending order of their contribution towards malaria research and the number represent percentage of their publications out of total published records. *NIH* National Institute of Health, *LSHTM* London School of Hygiene Tropical Medicine, *CNRS* The French National Centre for Scientific Research, *CDC* Centre for Disease Control Prevention, *RIIP* Réseau Institut Pasteur International, *US DOD* United States Department of Defense, *LSTM* Liverpool School of Tropical Medicine, *KEMRI* Kenya Medical Research Institute, *SWISS TPH* Swiss Tropical and Public Health Institute

The contributions from various organizations have been widely dispersed. However, the top contributing organizations have been depicted in Fig. 8A, B, which contributed between 1 and 5% of total publications. As evident from WOS (Fig. 8A), the University of London published 5,300 (~ 5%) records which is the highest amongst all other organizations. The National Institutes of Health (NIH; n = 4923), the London School of Hygiene & Tropical Medicine (LSHTM; n = 4333) and University of Oxford (UoO; n = 4276) contributed ~ 4% of total publications, each. The University of California contributed 3159 (~ 3%) publications. Other organizations from the top ten ranked list contributed < 3% each. One of the largest fundamental science agencies in Europe, the French National Centre for Scientific Research (CNRS), contributed ~ 2% (n = 2685) publications on malaria. Mahidol University (n = 2,653) and the Centers for Disease Control and Prevention (CDC; n = 2291) also contributed ~ 2% each. When the Scopus® database was examined, sequential variations were observed. The top three contributing organizations were the UoO with 5485 publications (~ 5%), the NIH with 4560 (~ 4%) publications and the LSHTM with 4,334 publications (~ 4%). Furthermore, the Mahidol University, the CDC, and the Liverpool School of Tropical Medicine (LSTM) published 2917, 2292, and 2257 records, respectively with a contribution of between 2 and 3%, each. Other organizations from the top-ten list contributed below 2% (Fig. 8B).

## Discussion

This analysis used three major scientific databases Web of Science^™^, Scopus^®^, and Embase^®^ to quantify the malaria-related publications in terms of various metrics. The three databases used in this study not only have wide coverage of articles but also have differential and complementary features. It has already been established that a reasonably good recall or coverage from a database search is not linearly associated with the number of databases searched but depends on the optimal use of selected databases [23]. SCO, launched in 2004, offers about 20% more coverage and covers a wider journal range than WOS which initially started in 1963 but WOS covers older publications as its search period goes as back as 1900 as compared to SCO (1966 to present) [25, 26] and therefore the combination of SCO and WOS offer the widest and oldest publication records in a scientific field. As EMB has additional focus and relevance to clinical sciences, its addition to SCO and WOS adds more depth to clinical literature search [23, 27]. Although it is suggested that a combination of EMB, PubMed, WOS, and Google Scholar offer a near-100% overall recall [23], PubMed and Google Scholar were replaced with SCO as the latter offered a similar/wider journal collection and the former two did not offer pre-analysed data output. However, EMB, SCO, and WOS are not freely available and need a paid subscription and, therefore, the analyses were dependent on the information that was available during the limited-period free-trial access (offered in 2020).

A near-identical rising trend of global malaria publications was noted with a slight dip between 2015 and 2020 in all three databases. The rising trend might indicate a constant researchers' interest, opportunities, funding, and need for malaria research globally whereas a dip may indicate a lack of commitment and complacency due to declining malaria cases around the world. This trend is more conspicuous in Fig. 6 where it can be reasonably debated that a sharp decrease in malaria cases between 2010 and 2014 might have translated into a decline in funding for malaria research and therefore leading to a decrease in the number of publications between 2015 and 2020 as noted in this analyses as has been reported by multiple authors in different areas [28, 29]. However, a spurt in the number of malaria cases globally from 2015 and more recently after the COVID-19 [24, 30], a concomitant response from all stakeholders to enhance commitment to malaria research funding and interest should drive up the annual number of malaria publications past the 2012–2015 levels.

In terms of the top 10 countries publishing most in malaria standardized by their respective population, only The Gambia and Gabon belonged to the malaria high-endemic regions. Nigeria, the Democratic Republic of Congo, and Uganda, the top 3 countries contributing the highest to malaria cases in 2020 [30] were ranked 69th & 59th, 87th & 100th, and 43rd & 40th, respectively in WOS & SCO in the total number of malaria publications across the period. Similarly, India, which contributes the highest number of cases in Southeast Asia, was ranked 84th & 80th, respectively in WOS and SCO. Although how a country’s contribution is counted in both databases may differ, it is evident that malaria-endemic countries do not publish much on malaria as compared to the countries with minimal or no malaria. Since the Malaria Journal is the only journal that is 100% dedicated to malaria-related publications, it is no doubt the topmost journal in terms of the number of publications as reported in the three databases analysed.

## Limitations

This publication analyses excludes the reports published in languages other than English and therefore underestimates the contribution of malaria-related publications in other languages such as French, Chinese, Spanish, German and Russian that contributed to ~ 1% of the publications each between 1945 and 2021, except French (~ 3%). Unlike pre-classified data, which had separate datasheets for each parameter alongside the number of publications, the raw data had all of the information in a single datasheet, including the year of publication, affiliation of authors, country, journal. The sole drawback of pre-classified data was that one could never correlate one statistic with another (for example, the year-wise performance of a country) because it provides pre-analysed data individually for each parameter. Therefore, to examine two parameters at once, raw data were utilized. A significant overlap was observed when analysing and categorization of publications according to the subject areas evidently due to multi-disciplinary research and lack of watertight compartmentalization between different scientific disciplines, for example, medicine and immunology or pathology and microbiology. Another limitation was encountered when compiling the affiliation at institutional level as different databases use different systems for classifying an institute.

## Conclusion

This research concludes a disproportional contribution of malaria research and publications between endemic and non-endemic countries with many times higher number of publications from non-malaria endemic countries. Because each of the explored databases had a different format for presenting the pre-analysed data, the output from different databases needs to be standardized so that a valid comparison could be made.

## Data Availability

Available on reasonable request.

## Abbreviations

RT: Research Translation
EMB: Embase
SCO: Scopus
WOS: Web of Science
LSHTM: London School of Hygiene & Tropical Medicine
CDC: Centers for Disease Control and prevention
UoO: University of Oxford
