# Correction: 75 years’ journey of malaria publications in English: what and where?

**DOI:** 10.1186/s12936-024-05048-0

**Published:** 2024-08-05

**Authors:** Nimita Deora, Sonalika Kar, Veena Pande, Abhinav Sinha

**Affiliations:** 1 ICMR-National Institute of Malaria Research, New Delhi, India; 2 Kumaun University, Nainital, India; 3 Academy of Scientific and Innovative Research, New Delhi, India

**Correction: Malaria Journal (2024) 23:172** 10.1186/s12936-024-04992-1

Following publication of the article [1], it came to the authors’ attention that the following Acknowledgements declaration had been omitted in error: “ICMR-National Institute of Malaria Research, New Delhi is acknowledged for infrastructural, administrative, and financial support.” The article has since been updated with this declaration. The authors thank you for reading this erratum and apologize for any inconvenience caused.
